# Narrative Review of Endodontic Biomaterials

**DOI:** 10.3390/biomimetics11030179

**Published:** 2026-03-03

**Authors:** Rosana Farjaminejad, Samira Farjaminejad, Alexander Garcia-Godoy, Franklin Garcia-Godoy

**Affiliations:** 1 Department of Health Services Research and Management, School of Health and Psychological Sciences, City University of London, London WC1E 7HU, UK; samira.farjaminejad@city.ac.uk; 2 Department of Bioscience Research, University of Tennessee Health Science Center, 7737 N University Dr, Ste 204, Tamarac, FL 33321, USA; agodoy2747@gmail.com; 3 Department of Bioscience Research, Bioscience Research Center, College of Dentistry, University of Tennessee Health Science Center, 875 Union Avenue, Memphis, TN 38163, USA; fgarciagodoy@gmail.com

**Keywords:** endodontics, biomaterials, bioceramics, regeneration, drug delivery

## Abstract

Advancements in biomaterials have transformed the field of endodontics, shifting treatment approaches from mechanical interventions to biologically driven regenerative therapies. This narrative review explores the evolving landscape of endodontic biomaterials, emphasizing their roles in disinfection, obturation, root repair, surgical procedures, and regenerative endodontics. Key materials such as mineral trioxide aggregate (MTA), Biodentine, and calcium-enriched mixture (CEM) cement demonstrate superior sealing, biocompatibility, and osteogenic potential compared to traditional materials. The integration of nanotechnology, bioactive components, and smart drug delivery systems has further enhanced antimicrobial properties and tissue interaction. Clinical applications, including regenerative procedures using platelet-rich fibrin and case-based biomaterial usage, are discussed to illustrate their relevance and effectiveness in real-world practice. Despite significant progress, challenges such as regulatory hurdles, economic limitations, and translational gaps persist. Emerging trends such as 3D printing, personalized medicine, and multifunctional scaffolds offer promising directions for future endodontic care. Continued interdisciplinary collaboration is essential to overcome current barriers and facilitate widespread adoption of next-generation biomaterials. Unlike prior reviews that categorize endodontic biomaterials descriptively by material class or technological advancement, this review introduces an indication-based comparative framework aligning biomaterial properties with specific clinical decision points and corresponding levels of evidence. By integrating biological mechanisms, translational considerations, and clinical application within a structured decision-oriented model, the manuscript offers analytical synthesis rather than a purely descriptive overview.

## 1. Introduction

The field of endodontics has undergone a remarkable transformation with the integration of advanced biomaterials that promote biocompatibility, tissue regeneration, and clinical success. Traditional materials such as gutta-percha and zinc oxide–eugenol sealers, though widely used, exhibit limitations like poor bioactivity, suboptimal sealing ability, and minimal interaction with surrounding tissues [1].

The development of bioactive and bioceramic materials, particularly calcium silicate-based cements such as MTA and Biodentine, has significantly enhanced endodontic treatment outcomes by offering improved sealing, antimicrobial properties, and the ability to support healing [2]. These materials exhibit strong interactions with periradicular tissues and stem cells such as periodontal ligament stem cells (PDLSCs), further supporting their use in biologically based procedures [3].

In regenerative endodontic procedures (REPs), scaffolds like platelet-rich fibrin (PRF) and blood clots play a critical role in enabling root development and tissue formation in immature teeth [4,5]. These scaffolds provide a matrix that supports stem cell differentiation and vascularization, while coronal barriers made from calcium silicates ensure isolation and promote healing [6].

Despite these advances, limitations persist. Common concerns include discoloration caused by MTA, handling challenges, extended setting times, and variable antibacterial efficacy [1]. Moreover, emerging materials such as CEM cement and modified MTA with additives like propylene glycol have shown potential improvements in sealing ability and biocompatibility during procedures like internal bleaching [7].

As the field advances, the role of stem cell interactions and nanotechnology in enhancing the physical and biological properties of endodontic biomaterials continues to be explored [3]. The inclusion of materials like bioactive glass, nanosilver particles, and radiopaque additives is guiding the next generation of restorative approaches [8]. Understanding these biomaterials in both clinical and biological contexts is essential for improving patient outcomes.

This review provides a consolidated analysis of key endodontic biomaterials used in irrigation, sealing, root repair, surgical applications, and regenerative therapy. It examines their physical, chemical, and antibacterial properties while presenting a clinical case to contextualize their application in real-world practice. While numerous reviews have discussed endodontic biomaterials according to material composition or emerging technological trends, few integrate biological mechanisms, specific clinical indications, and evidence hierarchy into a unified analytical structure. This review therefore introduces an indication-based comparative framework that maps material properties to defined treatment scenarios such as pulp capping, apexification, perforation repair, surgical endodontics, and regenerative procedures to enhance interpretive clarity and support evidence-informed clinical decision-making. As a narrative review, this manuscript is intended to provide an integrative and educational synthesis across clinical domains rather than a systematic evidence update or quantitative comparative analysis.

## 2. Literature Search Strategy

This narrative review was informed by a structured search of PubMed, Scopus, and Web of Science databases, focusing on publications from 2010 to January 2026. Keywords included “endodontic biomaterials,” “bioceramics,” “MTA,” “Biodentine,” “CEM cement,” “irrigation,” and “regenerative endodontics.”

Studies were selected based on their relevance to biomaterial properties, biological mechanisms, clinical application, and translational considerations. Both experimental and clinical studies were considered to provide a comprehensive interpretive synthesis rather than a systematic quantitative analysis.

## 3. Bioactive and Bioceramic Materials in Endodontics

### 3.1. Calcium Silicate-Based Bioceramics: Biological Rationale

Bioactive and bioceramic materials, particularly calcium silicate-based cements (CSCs), have shifted endodontic therapy from passive sealing toward biologically interactive treatment. Their clinical relevance extends beyond chemical composition to indication-specific performance, including pulp preservation, root-end sealing, perforation repair, and regenerative procedures. Therefore, these materials must be evaluated not only by physicochemical properties but also by clinical context and strength of supporting evidence.

Traditional obturation materials such as gutta-percha and zinc oxide–eugenol (ZOE)-based sealers provide adequate mechanical sealing but lack bioactivity and regenerative potential. In contrast, CSCs release calcium ions and create an alkaline environment that promotes hydroxyapatite formation, enhances sealing at the dentin interface, and supports hard tissue deposition. These biological interactions underpin their expanded clinical indications [1].

**Figure 1:**
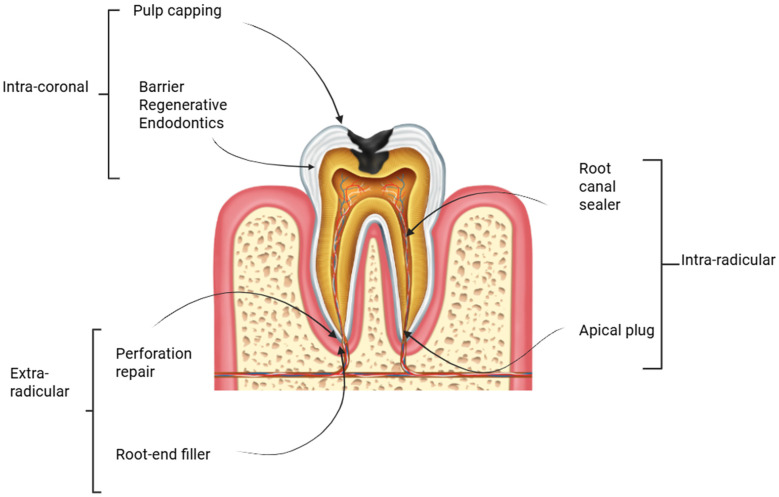
Clinical applications of bioceramic materials in endodontics categorized by anatomical site: intra-coronal (e.g., pulp capping, regenerative barriers), intra-radicular (e.g., sealers, apical plugs), and extra-radicular (e.g., perforation repair, root-end filling).

Figure 1 illustrates the various clinical applications of bioceramic materials in endodontics, categorized by anatomical location namely intra-coronal, intra-radicular, and extra-radicular uses.

### 3.2. Mineral Trioxide Aggregate (MTA)

Calcium silicate-based cement (CSC) represents the most frequently used class of bioactive materials in endodontics. ProRoot MTA, introduced in 1993, remains the benchmark bioceramic due to its sealing ability, antimicrobial behavior associated with high pH, and osteoinductive potential [9,10]. Its composition, based on tricalcium and dicalcium silicate, supports its use in a variety of applications including pulp capping, apexification, perforation repair, and retrograde fillings [11].

Commercial alternatives such as MTA Angelus have also gained clinical use; however, limitations including extended setting time, potential tooth discoloration, and handling difficulties have been reported [12]. These practical challenges have contributed to the development of newer calcium silicate-based formulations.

Biodentine, Bio-C Repair, and EndoSequence were developed to improve handling characteristics and address some of the limitations associated with earlier formulations. Biodentine, described as a tricalcium silicate-based dentin substitute, demonstrates improved mechanical properties and handling compared with traditional formulations. Bio-C Repair, a premixed silicate-based cement, has shown antibacterial activity and favorable cell viability in experimental investigations. EndoSequence has been associated with matrix metalloproteinase (MMP-2 and MMP-9) inhibition, which may contribute to modulation of pulpal inflammation; however, much of this evidence remains laboratory-based [13].

TheraCal LC, a light-cured material containing resin-modified calcium silicate components, combines bioactivity with enhanced handling properties. Its clinical application is primarily limited to pulp capping and vital pulp therapy rather than obturation. Current support for its use is largely derived from laboratory and short-term clinical findings [13]. These materials may incorporate components such as calcium phosphate, hydroxyapatite, bioactive glass, zirconia, and alumina, which contribute to radiopacity, mechanical reinforcement, and biointegration [1].

The concept of bio-obturation using CEM cement further illustrates the biologically oriented application of calcium silicate-based materials. In a 14-case report by Asgari (2025), CEM cement was applied in cases of internal and external resorption, perforations, and open apices, with reported symptom resolution and radiographic healing [14]. However, as the available evidence is primarily case-based, definitive comparative conclusions relative to MTA require further high-level controlled clinical studies.

Although MTA, Biodentine, and CEM cement share core bioactive characteristics, their clinical evidence profiles differ. MTA remains the most extensively studied material, particularly in pulp capping and surgical endodontics. Biodentine demonstrates favorable short-term clinical performance with improved handling characteristics, whereas CEM cement shows promising outcomes in perforation repair but is supported largely by case-based literature. These distinctions underscore the importance of aligning material selection not only with bioactivity but also with clinical indication and evidence hierarchy.

## 4. Endodontic Irrigation and Disinfection Materials

Effective irrigation is a fundamental component of successful endodontic treatment, playing critical mechanical, chemical, and microbiological roles in the elimination of biofilm, necrotic tissue, and debris from areas unreachable by mechanical instruments [15]. The apical third of the root canal system poses challenges due to anatomical complexity, making irrigation techniques and solution selection pivotal for clinical success.

### 4.1. Conventional Irrigants: NaOCl, EDTA, and CHX

Sodium hypochlorite (NaOCl) remains the most widely used irrigant due to its strong antimicrobial action and ability to dissolve organic tissue. However, NaOCl exhibits significant cytotoxicity when extruded into periapical tissues, which necessitates careful concentration control and delivery technique [15]. Ethylenediaminetetraacetic acid (EDTA), a chelating agent, is commonly employed as a final rinse to remove the smear layer and open dentinal tubules. Although it lacks strong antimicrobial properties, EDTA enhances the release of growth factors embedded within dentin and helps reduce the cytotoxic impact of NaOCl [16].

Chlorhexidine (CHX), a broad-spectrum antimicrobial agent, is often used as an adjunct or final irrigant due to its substantivity—i.e., prolonged antimicrobial activity on dentin surfaces. However, CHX does not dissolve organic material and can form a precipitate when mixed with NaOCl, raising safety concerns [15]. Alexidine (ALX), another bisbiguanide compound, has been comparatively evaluated against CHX in in vitro biofilm models. In a bioluminescence-based study assessing antibacterial efficacy against *Enterococcus faecalis*, 2% ALX demonstrated significantly lower residual bacterial activity than 2% CHX (14.20 ± 4.05 vs. 28.50 ± 6.62 relative luminescence units; *p* < 0.008), suggesting superior antimicrobial performance under the tested conditions [17]. This enhanced efficacy has been attributed to ALX’s ethylhexyl end groups, which facilitate deeper membrane lipid penetration and stronger electrostatic interaction with bacterial cell membranes [17].

### 4.2. Acidic Chelators and Pain Outcomes

Recent clinical trials have investigated the use of alternative chelators such as citric acid and etidronics acid (HEDP) in regenerative endodontic treatments (RETs). In a randomized clinical trial, canals irrigated with NaOCl followed by EDTA or citric acid showed significantly lower postoperative pain than those treated with NaOCl and HEDP. Although HEDP allows for simultaneous use with NaOCl without compromising its effectiveness, it was associated with increased postoperative discomfort [16].

Citric acid, known for its lower toxicity and high chelating efficiency, also promoted a higher release of TGF-β1 from dentin compared to EDTA, suggesting a favorable role in promoting regeneration in RETs [18]. These findings highlight the need to balance chelation strength, cytocompatibility, and biological signaling potential when selecting irrigants in regenerative procedures, particularly in light of emerging clinical outcome data.

### 4.3. Irrigant Activation and Delivery Techniques

Advances in irrigant delivery methods, including negative pressure systems like EndoVac, and ultrasonic activation devices, have aimed to enhance the efficacy and safety of irrigation protocols [19]. EndoVac facilitates deep penetration of irrigants into the apical third by delivering solutions under negative pressure, thereby minimizing the risk of extrusion and improving cleanliness in apical regions. In an in vitro study evaluating NaOCl, CHX, and ALX under both syringe and EndoVac delivery, NaOCl consistently demonstrated the highest reduction in bacterial load, regardless of delivery method. Although ALX showed lower residual bacterial activity than CHX under certain delivery conditions, negative-pressure irrigation did not significantly enhance the antibacterial efficacy of ALX compared to syringe irrigation, suggesting that irrigant chemistry may exert a greater influence on disinfection outcomes than delivery system alone [20,21].

Ultrasonic and sonic agitation have also been shown to increase the penetration and activity of irrigants, especially in isthmuses and lateral canals that are inaccessible to needle irrigation alone. These methods increase irrigant turnover, disrupt biofilm, and improve smear layer removal, supporting more predictable clinical outcomes [19].

### 4.4. Combination Irrigants and Emerging Trends

Combination irrigants such as QMiX, MTAD, and Tetraclean, which blend antimicrobial agents with smear layer-removing acids and surfactants, have been introduced to simplify irrigation protocols while reducing the risk of chemical interactions. These solutions are particularly useful for the final rinse phase, where they offer both antibacterial activity and chelating ability without the erosion risks associated with sequential NaOCl and EDTA use [15,19].

Additionally, nanoparticle-enhanced irrigants and herbal alternatives (e.g., propolis, curcumin) are gaining attention for their potential to offer lower toxicity and biofilm disruption without compromising antimicrobial efficacy [20,21,22]. Photodynamic therapy and laser-activated irrigation systems are also under investigation for improving bacterial eradication in resistant biofilms [23,24].

## 5. Surgical and Regenerative Endodontic Materials

Surgical endodontic procedures have evolved significantly, shifting from conventional apicoectomy toward modern endodontic microsurgery, which emphasizes precision, biological preservation, and enhanced healing outcomes. The aim of surgical intervention is not only to remove infected tissues and seal the root canal system but also to support periapical regeneration and retain the natural dentition whenever possible [25].

### 5.1. Root-End Filling Materials

A critical factor in surgical success is the choice of root-end filling material, which must provide a reliable apical seal, exhibit biocompatibility, and resist microbial leakage. While materials such as amalgam, glass ionomer cement (GIC), and zinc oxide–eugenol cements (e.g., Super EBA, IRM) have been used historically, they have demonstrated limitations including cytotoxicity, marginal leakage, and poor long-term stability [26].

The emergence of bioceramic materials, particularly MTA, has marked a turning point in retrograde filling. MTA’s favorable biological properties such as its ability to stimulate cementum formation and provide a moisture-tolerant seal make it a gold standard in modern apical surgery. More recently, materials like BioAggregate^®^, DiaRoot BioAggregate^®^, EndoSequence Root Repair Material (ERRM), and iRoot BP Plus have gained attention for their improved handling, enhanced radiopacity, and long-term sealing ability [27]. These materials are pre-mixed, injectable, and offer high alkalinity and bioactivity, making them well-suited for use in moist surgical environments.

Additionally, Geristore^®^, a resin-modified glass ionomer, has shown favorable adaptation to dentin and is sometimes used for subgingival repairs due to its strong adhesion and fluoride release. However, unlike calcium silicate-based materials, it lacks inherent bioactivity and may be more suitable in select periodontal-endodontic cases rather than in standard apical surgery [28].

A clinical comparison of root-end filling techniques revealed that BioAggregate achieved faster and more predictable healing than traditional methods involving gutta-percha or resin-based fillers like Retroplast^®^, which, while adaptable, may lack the biological advantages of newer bioceramics [27].

### 5.2. Regenerative Materials in Endodontic Surgery

In complex periapical lesions, particularly those with large bone destruction, cortical plate involvement, or sinus communication, regenerative biomaterials are increasingly integrated into the surgical protocol. These include barrier membranes, bone graft substitutes, and autologous platelet concentrates (APCs). Barrier membranes, especially resorbable collagen-based membranes such as Bio-Gide^®^, serve to prevent soft tissue proliferation into osseous defects and allow osteogenic cell repopulation. Non-resorbable membranes like expanded polytetrafluoroethylene (e-PTFE) have also been used, although they typically require a second surgery for removal and carry a higher risk of exposure [29].

Bone grafts such as calcium phosphate ceramics, bovine-derived hydroxyapatite (e.g., Bio-Oss^®^), and demineralized freeze-dried bone allografts (DFDBA) act as osteoconductive scaffolds that support bone formation. However, unlike purely osteoconductive materials such as xenografts and synthetic ceramics, DFDBA also exhibits osteoinductive properties due to the exposure of bone morphogenetic proteins (BMPs) during the demineralization process, which can stimulate progenitor cell differentiation and new bone formation. In guided tissue regeneration (GTR), the combination of graft and membrane materials has been shown to improve clinical outcomes, particularly in through-and-through defects or when the buccal cortical plate is missing [30]. Autologous platelet concentrates, including platelet-rich fibrin (PRF) and platelet-rich plasma (PRP), are increasingly used to accelerate healing due to their release of growth factors like VEGF and TGF-β. PRF offers ease of preparation without anticoagulants and has shown benefits in both soft tissue healing and bone maturation [30].

The integration of these regenerative adjuncts enhances the biological potential of surgical endodontic treatment, not only improving healing but also reducing postoperative discomfort and enhancing long-term success. Their application should be tailored to the clinical scenario based on defect size, location, and patient-specific healing capacity.

## 6. Clinical Applications: Summary of Case Reports

Numerous clinical case reports and small case series have described the application of bioactive and bioceramic materials in a range of endodontic scenarios, including apexification, perforation repair, dens invaginatus management, regenerative procedures, and pulp preservation. These reports provide insight into clinical feasibility and short- to mid-term outcomes across diverse treatment contexts [Table 1].

It should be noted that the majority of the studies summarized are case reports or small case series, representing lower levels of evidence within the evidence hierarchy. Therefore, the findings presented illustrate clinical applications and reported outcomes rather than establish definitive comparative effectiveness among materials.

## 7. Material Properties and Performance of Endodontic Biomaterials

The clinical efficacy of endodontic materials hinges on a balance of physical robustness, biological compatibility, and antibacterial action. These properties directly influence sealing ability, longevity, and the ability to promote periapical healing.

### 7.1. Physical Properties

Key physical characteristics such as setting time, compressive strength, solubility, and pH are critical for clinical usability. MTA, for instance, demonstrates strong sealing and dimensional stability; however, its extended setting time (up to 4 h) and granular consistency pose handling challenges. Biodentine, with a faster setting time (approximately 12–30 min), addresses this limitation through its calcium chloride-accelerated formulation and enhanced powder–liquid interaction, improving both manipulation and clinical workflow [48].

Premixed calcium silicate cements (pCSCs), utilizing vehicles like polyethylene glycol (PEG), propylene glycol (PG), or dimethyl sulfoxide (DMSO), further influence setting dynamics. For example, PG-based cements exhibit superior compressive strength and lower solubility compared to PEG-based variants. Biodentine, specifically, maintains higher compressive strength post-irrigation when compared to NaOCl-exposed or EDTA-treated specimens, indicating better mechanical integrity under clinical conditions [48].

### 7.2. Biological Properties

Biological performance encompasses biocompatibility, bioactivity, and long-term integration. MTA and other tricalcium silicate-based cements elicit minimal inflammation and promote cementogenesis and osteogenic differentiation by releasing calcium ions and maintaining high alkalinity (pH > 11) during hydration. These materials also support the formation of hydroxyapatite at the interface with dentin, reinforcing their role in pulp capping, apexification, and root repair. In vivo studies have demonstrated the ability of materials like BioAggregate and MTA Repair HP to stimulate hard tissue formation and avoid foreign body responses, particularly in regenerative procedures and surgical applications [49,50].

### 7.3. Antibacterial Properties

Endodontic biomaterials should inhibit bacterial proliferation and resist biofilm formation, especially against persistent species such as *Enterococcus faecalis*. MTA’s high initial pH contributes to its antibacterial effect, though it lacks active bactericidal agents. Bioceramic sealers and modified materials, such as Bio-C Repair and EndoSequence, demonstrate superior antimicrobial activity, with enhanced resistance to microbial infiltration when paired with appropriate irrigation regimens. Notably, materials like TheraCal LC, while designed primarily for pulp capping, incorporate resin-modified components to enhance sealing and antibacterial behavior. However, their long-term behavior and interaction with dentin substrates require further clinical validation [51].

### 7.4. Indication-Based Comparative Framework

To further operationalize the proposed indication-based comparative framework, the following table maps common endodontic clinical scenarios to representative biomaterials and summarizes the predominant type of supporting evidence cited in this review. This structured overview is intended to enhance clinical interpretability and strengthen the decision-oriented synthesis of the available literature [Table 2].

The characterization evidence reflects the predominant study designs cited within this narrative review and is intended to provide interpretive guidance rather than formal evidence grading.

## 8. Challenges and Future Directions

Despite considerable advancements in endodontic biomaterials, several challenges remain that hinder full clinical integration, long-term success, and widespread accessibility.

### 8.1. Translational Barriers and Clinical Trial Limitations

A primary obstacle in adopting novel endodontic biomaterials is the gap between laboratory innovation and clinical validation. While many materials demonstrate promising in vitro performance, relatively few are supported by long-term, well-designed randomized clinical trials. Variability in composition, non-standardized testing protocols, and limited follow-up periods hinder robust comparative conclusions regarding durability and clinical effectiveness [52,53].

Additionally, regulatory pathways for dental biomaterials differ globally and require extensive documentation of safety and biocompatibility. This process, although essential, contributes to delays in translation from experimental development to routine clinical use [54].

### 8.2. Regulatory and Economic Considerations

The increasing use of advanced biomaterials such as MTA, Biodentine, and BioAggregate in endodontics has brought significant improvements in biological performance and clinical outcomes. However, these materials are associated with high production costs and limited affordability in many clinical settings. For instance, ProRoot MTA and MTA Angelus are frequently reported as relatively higher-cost calcium silicate-based materials, whereas Biodentine and TheraCal LC are comparatively more affordable depending on regional availability and market context [1].

Beyond direct material costs, advanced formulations—particularly those incorporating nanoparticle modifications—require additional manufacturing controls and regulatory validation. While such modifications may enhance antimicrobial or mechanical performance, they increase production complexity and extend approval processes [1,14,55].

These economic and regulatory factors may limit accessibility, especially in public healthcare systems and developing regions where reimbursement policies may not align with emerging biomaterial technologies. Addressing these barriers will require coordinated efforts among clinicians, researchers, manufacturers, and regulatory authorities to standardize evaluation frameworks and improve global access to evidence-based endodontic materials [1,56].

### 8.3. Innovative Trends and Future Technologies

Ongoing research is focused on the development of multifunctional, smart biomaterials that can adapt to their biological environment [57]. Nanotechnology plays a pivotal role by enhancing not only the antibacterial and sealing properties but also promoting tissue regeneration. Materials integrated with silver, zinc oxide, and carbon-based nanoparticles exhibit superior mechanical strength and bioactivity while resisting microbial colonization [58,59].

Another promising direction is 3D printing of patient-specific scaffolds for regenerative endodontic procedures [60]. These constructions, often fabricated using bio-inks that contain calcium silicates or hydrogels with stem cells, offer tailored geometry and biofunctionality for apexogenesis and tissue engineering [61,62].

Drug-delivery-enabled biomaterials, such as those incorporating montmorillonite or nano-hydroxyapatite, are also under investigation. These systems could allow for the localized, sustained release of antibiotics, anti-inflammatory agents, or growth factors within the root canal system [63].

### 8.4. Personalized and Biologically Driven Approaches

The paradigm in endodontic therapy is gradually shifting from a purely mechanistic intervention toward biologically driven and patient-tailored strategies [64]. This transition is supported by the emergence of precision endodontics, which integrates clinical imaging, risk assessment tools, and biomaterial innovations to optimize treatment outcomes for individual patients [60].

Digital dentistry, including CBCT and intraoral scanning, has enabled the development of guided endodontics, a technique particularly beneficial for cases involving pulp canal obliteration (PCO) or calcified canals. By merging CBCT datasets with intraoral scans, clinicians can digitally plan the canal access path and fabricate 3D-printed endodontic guides. This approach, as demonstrated by Dąbrowski et al. (2022), allows for minimally invasive root canal access while preserving pericervical dentin, a critical structure for fracture resistance and long-term tooth prognosis [65].

In parallel, personalized medicine in endodontic–periodontal lesions (EPLs) focuses on risk-based treatment decisions. As outlined by Takahashi et al. (2022), EPLs present multifactorial pathogenesis involving microbial, anatomical, and systemic factors, requiring individualized diagnostic algorithms [66]. Modern classifications of EPLs now incorporate iatrogenic errors, patient-specific risk factors (e.g., diabetes, smoking), and tissue healing capacity, offering a comprehensive basis for tailored therapeutic strategies.

These advances not only improve diagnostic accuracy but also reduce treatment-associated complications and enhance healing. The combination of smart biomaterials, computational modeling, and patient-specific data is expected to refine prognosis prediction and support biologically conservative endodontic interventions [67,68].

### 8.5. Priority Research Questions and Near-Term Directions

It is important to acknowledge that a substantial proportion of the currently available literature on newer endodontic biomaterials consists of case reports and small case series. While these publications provide valuable clinical insight and illustrate feasibility across diverse treatment scenarios, they represent lower levels of evidence within the evidence hierarchy. Consequently, definitive comparative conclusions regarding material superiority or long-term effectiveness remain limited by the predominance of case-based data.

To strengthen translational impact, future investigations should prioritize clearly defined and testable research objectives. Near-term studies may focus on: (1) randomized controlled clinical trials comparing calcium silicate-based materials with standardized follow-up periods of at least 24–36 months to evaluate long-term clinical performance; (2) in vivo assessment of nanoparticle-enhanced biomaterials to determine cytocompatibility thresholds, inflammatory response, and antimicrobial durability; (3) quantitative evaluation of drug-eluting endodontic materials, including release kinetics and periapical tissue integration; and (4) comparative clinical studies assessing the effect of guided endodontics and 3D-printed scaffolds on procedural accuracy, dentin preservation, and fracture resistance. Establishing standardized outcome measures and multicenter collaborations will be essential to generate reproducible and clinically meaningful evidence. Collectively, these limitations highlight that material selection in endodontics should be guided not only by biological properties but also by indication-specific evidence strength, long-term clinical performance, economic feasibility, and practical handling considerations.

## 9. Conclusions

The integration of advanced biomaterials into endodontic practice has significantly enhanced clinical outcomes, transforming the field from traditional mechanical approaches to biologically driven, regenerative therapies. Materials such as MTA, Biodentine, and CEM cement have proven effective in promoting periapical healing, supporting tissue regeneration, and providing long-term sealing, particularly in challenging cases like immature teeth, root resorptions, and perforations.

This review highlighted the broad scope of endodontic biomaterials from irrigation and obturation to surgical and regenerative applications. The physical, chemical, and antibacterial properties of these materials underpin their success, while bioceramics and bioactive formulations continue to evolve with the integration of nanotechnology and smart delivery systems.

Despite their promise, barriers remain. Clinical adoption is limited by high costs, regulatory complexities, and a lack of long-term, high-quality clinical trials. Moreover, real-world variability in handling properties, cytocompatibility, and outcomes underscores the need for standardized testing and material characterization.

Future directions point toward personalized endodontics leveraging imaging technologies, computational modeling, and patient-specific biomaterials to achieve tailored treatment outcomes. Advances in 3D printing, bioactive scaffolds, and multifunctional nanocomposites offer exciting opportunities for precision, minimally invasive, and biologically aligned endodontic care.

Continued interdisciplinary collaboration among clinicians, material scientists, and regulatory bodies will be essential to bridge translational gaps and make these innovations accessible and sustainable in everyday dental practice. Given the narrative nature of this review, the conclusions presented aim to synthesize and contextualize current knowledge across clinical domains rather than provide a definitive quantitative evidence update.

## Figures and Tables

**Table 1:** Summary of selected cases from the literature, highlighting the diversity of biomaterials used and the corresponding clinical observations reported in individual cases.

Author and Year	Material Used	Condition Treated	Tooth Involved	Outcome	Study Design	Ref.
**Patankar et al. (2025)**	Platelet-Rich Fibrin (PRF)	Persistent periapical granuloma post-RCT	Maxillary first premolar & first molar	Radiographic bone fill at 6 and 12 months	Case report (2 cases)	[31]
**Nadgouda et al. (2024)**	Biodentine	Open apex	Maxillary central incisor	Apex closure and dentinogenesis (6-month follow-up)	Case report	[32]
**da Silva et al. (2025)**	Emdogain (Enamel matrix derivative)	Necrotic immature permanent teeth	Multiple teeth (aged 8–12)	Root lengthening, dentin thickening, apex closure (24-month follow-up)	Case series	[33]
**Alelyani (2024)**	Bio-C Repair	Immature tooth with necrotic pulp	Maxillary central incisor	Complete root formation and apical closure (12-month follow-up)	Case report	[34]
**Asgary (2024)**	Calcium-Enriched Mixture (CEM) Cement	Internal root resorption	Contralateral mandibular molars	Complete healing of apical lesions at 5 years	Case report	[35]
**DeMayo et al. (2025)**	Bioceramic fast-set putty	Dens evaginatus with necrosis	Mandibular second premolar	Apex closure and thickened root walls at 15 months	Case report	[36]
**Marra et al. (2024)**	Bioceramic sealer	Type III Dens Invaginatus	Maxillary lateral incisor	Radiographic healing with periapical repair (20-month follow-up)	Case report	[37]
**Priyadarshini et al. (2024)**	Endoflas vs. Endoflas with Curcumin Gel (EPCG)	Chronic irreversible pulpitis in primary molars	Mandibular molars 74 and 85	Successful pulpectomy, reduced inflammation (12-month follow-up)	Case report	[38]
**Hillary et al. (2024)**	Bio-C Repair	Immature tooth with open apex	Maxillary anterior tooth	Healing seen at 2 and 4 months	Case report	[39]
**Rezaei & Fazlyab (2025)**	Calcium-Enriched Mixture (CEM) Cement	Type IIIa Dens Invaginatus with chronic apical abscess	Maxillary central incisor	Resolution of lesion and cementogenesis at 15 months	Case report	[40]
**Sanyal et al. (2025)**	MTA, Biodentine	External Cervical Resorption with widened apex	Maxillary right central incisor	Tooth reinforced and sealed successfully with biomimetic restoration (18-month follow-up)	Case report	[41]
**Chamani et al. (2025)**	Bioceramic sealer, MTA	Root perforation in hypertaurodont molar	Maxillary second molar	Symptom-free function and radiographic healing at 12 months	Case report	[42]
**Suryawanshi et al. (2024)**	MTA, Fiber-Reinforced Composites (FRC)	Iatrogenic furcal perforation	Maxillary first molar (tooth #26)	Sealed and structurally reinforced; stable at 6 months	Case report	[43]
**Vidal et al. (2016)**	Biodentine	Apexification in immature permanent tooth	Tooth #9	Apical closure and absence of symptoms at 18 months	Case report	[44]
**Bhasin et al. (2024)**	MTA, Bioceramic Sealer	Open apex with blunderbuss canal	Tooth #11	Healing of periapical lesion and clinical success at 9 months	Case report	[45]
**Victorino et al. (2025)**	MTA Repair HP, Hemospon	Apexification in anterior tooth with incomplete root formation	Tooth #21	Complete periapical healing at 27 months	Case report	[46]
**Mazumdar et al. (2020)**	MTA, Activa Bioactive Composite Resin	Direct pulp capping in reversible pulpitis	Molars (Tooth #36 and #26)	Pulp vitality and function maintained at 6 months	Case report	[47]

**Table 2:** Indication-Based Comparative Framework Aligning Clinical Scenarios, Biomaterials, and Predominant Evidence Base.

Clinical Scenario	Commonly Used Biomaterial(s)	Predominant Evidence Base	Key Limitations
Vital pulp therapy/Pulp capping	MTA, Biodentine, TheraCal LC	Randomized and prospective clinical studies (MTA, Biodentine); limited clinical data for resin-modified materials	Some materials lack long-term follow-up; discoloration and handling variability
Apexification (immature teeth)	MTA, Biodentine, CEM cement	Clinical studies with substantial case-based literature	Limited high-level comparative trials
Perforation repair	MTA, Bio-C Repair, CEM	Primarily case reports and small case series	Limited long-term comparative evidence
Root-end filling (surgical endodontics)	MTA, BioAggregate, ERRM	Clinical studies and systematic reviews for MTA; fewer comparative trials for newer materials	Cost and variability in study protocols
Regenerative endodontic procedures (REPs)	PRF, MTA coronal barrier, scaffolds	Limited clinical trials with predominance of case-based evidence	Heterogeneous regenerative protocols
Nanoparticle-modified or experimental biomaterials	Various modified bioceramics	Primarily laboratory and preclinical studies	Limited human clinical validation

## Data Availability

No datasets were generated or analyzed during the current study.
